# Understanding Cognitive Deficits in People with Arthritis

**DOI:** 10.3390/healthcare11091337

**Published:** 2023-05-06

**Authors:** Weixi Kang, Edward Whelan, Antonio Malvaso

**Affiliations:** 1 Imperial College London, London SW7 2BX, UK; 2 Independent Researcher, 99MX QH Maynooth, County Kildare, Ireland; 3 Department of Brain and Behavioral Sciences, University of Pavia, 27100 Pavia, Italy

**Keywords:** arthritis, cognitive deficits, episodic memory, semantic verbal fluency, fluid reasoning, numerical ability

## Abstract

Cognitive deficits are commonly seen in people with arthritis. However, previous studies focused primarily on small-sized clinical samples. There is a need for cohort-based studies, which are characterized by high generalizability. In addition, these studies mainly focused on attention, memory, and executive function. However, cognition is not a single concept, but includes other cognitive domains, such as verbal fluency and arithmetic abilities. Thus, we aim to explore how arthritis can affect cognitive abilities, including episodic memory, semantic verbal fluency, fluid reasoning, and numerical ability by using a large cohort from the United Kingdom. The main findings were that people with arthritis have significantly lower immediate word recall (t(2257) = −6.40, *p* < 0.001, Cohen’s d = −0.12, 95% C.I. = [−0.16, −0.08]), delayed word recall (t(2257) = −5.60, *p* < 0.001, Cohen’s d = −0.11, 95% C.I. = [−0.14, −0.07]), semantic verbal fluency (t(2257) = −3.03, *p* < 0.01, Cohen’s d = −0.06, 95% C.I. = [−0.10, −0.02]), fluid reasoning (t(2257) = −3.96, *p* < 0.001, Cohen’s d = −0.07, 95% C.I. = [−0.11, −0.04]), and numerical ability (t(2257) = −3.85, *p* < 0.001, Cohen’s d = −0.07, 95% C.I. = [−0.10, −0.03]) compared to what they would expect given their demographics. Interventions are needed to improve cognitive abilities in people with arthritis.

## 1. Introduction

Cognitive deficits are usually described as problems with memory, learning, analytical ability, concentration, and decision-making [1]. Recent research has concluded cognitive functioning is affected by arthritis, which is a long-term disease characterized by joint inflammation. Indeed, the literature generally agrees that people with arthritis tend to have cognitive impairments [2,3,4].

Arthritis is a group of over 100 distinct conditions that affect the joints, cartilage, and other connective tissues of the body. The most common types of arthritis are non-inflammatory osteoarthritis, inflammatory rheumatoid arthritis, psoriatic arthritis, fibromyalgia, and gout [5]. Rheumatoid arthritis, a persistent inflammatory condition caused by the immune system’s own reaction, is characterized by symmetrical, progressive joint inflammation that erodes bone, damages cartilage, impairs mobility, and reduces quality of life [3]. Osteoarthritis of the hip joint is a chronic, irreversible condition that involves the progressive destruction of articular cartilage, making it the most disabling form of arthrosis with a significant impact on patients’ quality of life [4].

Preliminary research suggests that osteoarthritis may raise the risk of cognitive decline and dementia, potentially due to inactivity and a lack of exercise [6,7]. Recent studies have linked inflammatory factors to cognitive performance, revealing a connection between increased systemic inflammation and poor cognitive functions in middle-aged individuals [8]. Animal studies have also indicated that inflammation associated with osteoarthritis may promote Alzheimer’s disease pathology [9]. On the other hand, rheumatoid arthritis-related pain has been shown to negatively impact cognitive ability, particularly in the domains of attention and executive function, such as working memory and set shifting [10,11,12].

Bartolini et al. (2002) reported that arthritis patients often have problems with their cognitive functions and that there was a prevalence rate of 38% (attentional deficits and mental flexibility) to over 70% (impaired visuospatial ability and planning) in that population [13]. Dick et al. (2007) focused on attentional functioning in people with arthritis and compared it with those with a diagnosis of musculoskeletal (MSK) pain and fibromyalgia (FM). This study revealed that there are cognitive problems in all the samples [14], which provided backup assertations that chronic pain sufferers have considerable attention deficits. Ziarko et al. (2019) indicated that the severity of pain, management strategies, and personal resiliency are all influenced by the level of anxiety and depressive symptoms [15]. Dick and Rashiq (2007) investigated how pain affects attention and memory processes and discovered that two-thirds of chronic pain participants exhibit cognitive impairments, such as sustaining attention and preserving a memory trace throughout a working memory task [16].

One study found that arthritis patients with both pain and depressive symptoms all have poor performance on a cognitive test. When depression was a mediating factor, the influence of pain on cognitive functioning was minimal [17]. However, other research showed a negative association between performance and arthritis pain on a test that involved attention, control, and working memory [18].

Another study tested the cognitive capacities of three groups (healthy controls, arthritis patients, and FM patients), measuring their cognitive abilities. Global attention, linguistic processing, working memory, verbal and visual memory, executive function, and spatial processing were among them. This study found that both FM and arthritis patients scored low in many measures of cognitive performance when compared to healthy controls [19]. Differences in FM and arthritis patients’ cognitive functions were not significant based on their executive function performance. Cognitive symptoms may be increased if the patient is tired, has inadequate quality sleep, and is in pain. However, how these factors may relate to cognitive issues is not known.

Acute pain, recurrent pain, and chronic pain in the three groups of patients were also investigated [20], and three cognitive function assessments were used, including cognitive control, psychomotor ability, and sustained attention. Studies found that those with chronic pain had inferior performance concerning their psychomotor and attention functions compared to the healthy controls. Acute discomfort was significantly associated with decreased psychomotor performance. Those who have recurring episodes of pain have inferior performance when it comes to attentional performance [20]. Brain regions, including the anterior cingulate cortex, prefrontal cortex, and secondary somatosensory cortex, play a significant role in cognitive processing of a higher order, including concentration, cognitive control, and psychomotor performances [21,22]. Moore et al. (2012) used thermal pain to manipulate attention span, task switching, and dual attention [23]. This study showed that activities demanding higher-order processes, especially those related to executive control, are liable to be influenced by pain [24].

Understanding the effect of arthritis on cognitive abilities is important because inferior cognitive functions are associated with a resultant loss of physical functioning in everyday activities. Indeed, there is a clear link between cognitive functioning and physical performance [25], especially among elderly adults [26]. Shin et al. (2013) studied the link between cognitive and physical function in arthritis. The results of this study, which included 12 standardized neuropsychological tests, revealed that cognitive impairment was a role in increased functional limits in arthritic patients, and these reductions were confirmed in both self-reported and performance-based assessments [27]. Arthritis and its associations with cognitive difficulties can negatively impact everyday activities and functioning treatment management and coping with illness [27,28]. One study has shown that arthritis is not only a disease with levels of pain and dysfunction, but it also has a psychological dimension. For instance, pain is associated with arthritis in every way and has a negative impact on QoL (quality of life) [29].

Thus, although there are some studies that have identified cognitive deficits in people with arthritis, they focused primarily on small-sized clinical samples. There is a need for cohort-based studies, which are characterized by high generalizability. In addition, these studies mainly focused on attention, memory, and executive function. However, cognition should not be considered a unitary concept, but also includes other domains, such as numerical ability and verbal fluency. Thus, we aim to explore how arthritis can affect cognitive abilities, including episodic memory, semantic verbal fluency, fluid reasoning, and numerical ability.

## 2. Methods

### 2.1. Participants

Understanding Society: the UK Household Longitudinal Study (UKHLS) data were used, which have been collected as annual information from original sample houses since 1991 [30]. Those who participated were asked by researchers to answer if they received a diagnosis of arthritis clinically in Wave One (data collection from 2009 to 2010). Then, participants were also asked if they had been newly diagnosed with arthritis in each wave until Wave Three. Participants who indicated that they had been clinically diagnosed with arthritis at any time point were considered as people with arthritis, whereas the rest of the participants were considered as people without arthritis. Participants completed cognitive measures in Wave Three (data collected between 2011 and 2012). We eliminated individuals with missing data from the study. We also selected healthy controls matched by age and sex from those who stated that they had not received a diagnosis of arthritis clinically. A total of 2258 participants with a diagnosis of arthritis were included, with an average age of 61.18 ± 14.51, including 65.85% females, as well as 21.66% who received college-level education and 3792 individuals with no arthritis diagnosis, with an average age of 61.44 ± 9.95, including 65.88% females and 26.19% who received college-level education.

### 2.2. Instruments

Episodic memory can be measured with immediate and delayed word recall. An animal fluency task was employed to test the semantic verbal fluency of participants [31,32,33]. The number series task was used to examine fluid reasoning, which is the capacity to use abstract reasoning to solve unfamiliar problems and is usually assessed by means of logical games or puzzles [31]. We measured numeracy in a series of questions including “In a sale, a shop is selling all items at half price. Before the sale, a sofa costs £300. How much will it cost in the sale?” Details of these questions can be found at [34] and were copied below. All scores were standardized before further analysis.

- The immediate and delayed recall tasks: “For this task, the computer reads a list of 10 words to standardise the presentation and speed of the word list. The interviewer checks if the respondent can hear the computer playing a short test message. If the voice cannot be heard the interviewer checks again following adjustment of the volume. If the respondent still cannot hear the computer’s voice, the interviewer reads the words at a slow steady rate of about one word every two seconds. The list of words is not repeated. No aids are allowed for the test. Interviewer: The computer will now read a set of 10 words. I would like you to remember as many as you can. We have purposely made the list long so it will be difficult for anyone to remember all the words. Most people remember just a few. Please listen carefully to the set of words as they cannot be repeated. When it has finished, I will ask you to recall aloud as many of the words as you can, in any order. Is this clear? Now please tell me the words you can remember. Respondents give the words in any order. The interviewer codes each correct response. For the delayed word recall test, after the Number Series test (below), respondents were again asked to remember the words from the list. The interviewer codes each correct response. We used the word lists developed for the HRS, as does ELSA. The different lists were given to members of the same household based on random assignment. The lists can also be varied in subsequent waves to reduce learning. Table 1 has the word lists”.
- Animal naming task: “Interviewer: Now, I would like you to name as many animals as you can. You have one-minute, so name them as quickly as possible. We will begin when you say the first animal. If you are unsure of anything please ask me now as I am unable to answer questions once the minute starts The interviewer instructions are to write down the actual words in the order in which they are produced. They are recorded in the Cognitive Ability Booklet. With respect to scoring, extinct, imaginary or magical (e.g., dodo, unicorn, dragon) animals were scored as correct, but given names (e.g., Felix, Buster) were not. The assessment was timed by CAPI. The interviewer began the 60 s countdown on the computer as soon as the respondent said the first correct word”.
- Number series task: “For this test, respondents use a pencil and paper to write down the number sequences as read by the interviewer. The number series consists of several numbers with a blank number in the series. The respondent will be asked which number goes in the blank. The interviewer begins with a simple example so the respondent can see how the test works. For the example, the interviewer can tell the respondent if they give an incorrect response and inform them of the correct answer. If the respondent does not understand the instructions, or answered ‘Don’t know’ in the example, a further example is worked through. If they answer incorrectly a second time, CAPI instructs the interviewer to inform them of the correct response and explain how the sequence works. If the respondent still does not understand, or seems confused, the interviewer codes this and moves on to the next task. However, if the respondent understands the task, the interviewer moved on to the number series”.
- Numerical ability test: “Interviewer: Next I would like to ask you some questions to understand how people use numbers in everyday life. If CATI, the interviewer added, You might want to have a pencil and paper handy to help you answer the following items The measure of numeric ability asks respondents up to five questions that are graded in complexity. Based on performance on the first three items, respondents can get additional more difficult items and a higher score or an additional more simple item. ‘Don’t know’ was not a permitted response. There was a showcard with the text of the question. This can be seen in the fieldwork documents” [34].

### 2.3. Demographic Controls

Age (continuous), sex, highest educational qualification, legal marital status, monthly income (continuous), and residence were all demographic controls in the model. Please refer to Table 1 in terms of how they were coded.

### 2.4. Analysis

All cognitive measures were first standardized. We used a predictive normative modeling approach to analyze data. First, three generalized linear models were constructed by taking demographics, which were included as predictors to predict cognitive measures in healthy controls. Then, these models were applied to people with arthritis to predict their expected cognitive scores, imagining that they are healthy. Finally, we used one-sample t-tests to examine the differences between predicted and actual cognition scores in patients with arthritis. Because it can control for demographic factors and deal with uneven sample size, this predictive normative modeling approach outperforms the paired sample t-test.

## 3. Results

**Figure 1:**
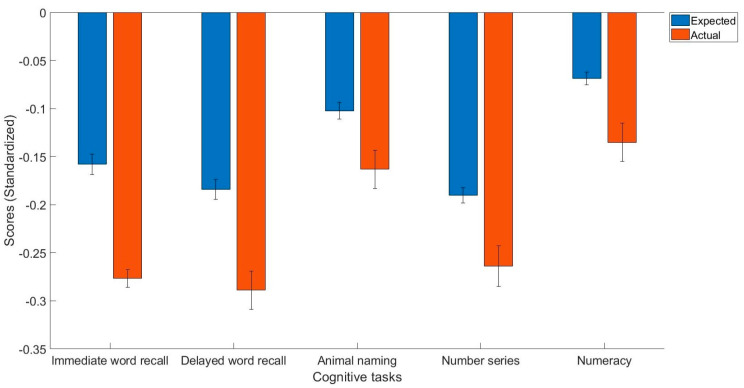
The expected and actual cognitive task scores in people with arthritis.

Table 2 contains descriptive statistics. Table 3 and Table 4 contain the results of general linear models trained on healthy controls. The main findings were that people with arthritis have significantly lower immediate word recall (t(2257) = −6.40, *p* < 0.001, Cohen’s d = −0.12, 95% C.I. = [−0.16, −0.08]), delayed word recall (t(2257) = −5.60, *p* < 0.001, Cohen’s d = −0.11, 95% C.I. = [−0.14, −0.07]), semantic verbal fluency (t(2257) = −3.03, *p* < 0.01, Cohen’s d = −0.06, 95% C.I. = [−0.10, −0.02]), fluid reasoning (t(2257) = −3.96, *p* < 0.001, Cohen’s d = −0.07, 95% C.I. = [−0.11, −0.04]), and numerical ability (t(2257) = −3.85, *p* < 0.001, Cohen’s d = −0.07, 95% C.I. = [−0.10, −0.03]) compared to what they would expect given their demographics (Figure 1).

## 4. Discussion

Taken together, we aimed to explore how arthritis could affect cognitive abilities using immediate word recall, delayed word recall, semantic verbal fluency, and numeracy tasks. The results showed that people with arthritis have poorer performance in all these cognitive tasks. This study provided novel findings, including that many cognitive domains, such as episodic memory, semantic verbal fluency, fluid intelligence, and numerical abilities, are negatively affected by arthritis in a cohort-based study based on participants from the United Kingdom. Yet, their differences were small, which are not equivalent to cognitive impairments per se. However, they may still have an impact on quality of life [3] and could, in the long run, lead to cognitive impairments.

Several potential pathways may explain poor cognitive performance in people with arthritis. Prior studies have shown that chronic pain with arthritis interferes with concentration, which causes significant impairments in daily functioning and lower quality of life. According to prevalence studies, up to 44% of the population experiences pain on a regular basis, with one-quarter experiencing severe pain [35,36]. Many people with persistent pain also have attention and other deficits. Pain can transmit warnings of real or potential pain [17]. Those who suffer chronic pain experience disruption to their memory and attention [37]. Chronic pain can impact every aspect of their life, such as sleep, which can become progressively worse. Research by Eccleston and Crombez (1999) found that chronic pain patients who self-reported elevated levels of both pain and bodily awareness have a decline in performance when performing demanding tasks, including the switching task [38]. The reason for this relationship between pain and cognitive/attention function is not really known, but it is probable that the common underlying neural basis is a factor, as similar attentional resources are required for pain, just as other mental functions.

Second, treatments routinely used to treat pain in arthritis patients can adversely influence their mental abilities. Corticosteroid is responsible for limiting the performance of their memory. Wolkowitz et al. (1990) reported that a single dosage (e.g., 1 mg of dexamethasone) or short-term corticosteroid usage (e.g., 80 mg of prednisone over five days) could be related to memory problems [39].

Third, psychiatric conditions associated with arthritis may result in poor cognitive performance. For instance, Smedstad et al. (1996) has shown that arthritis symptoms can exacerbate psychiatric illnesses [40]. Covic et al. (2006) reached similar conclusions that the level of fatigue and pain in arthritis can predict depressive symptoms [41]. However, Huyser et al. (1998) only confirmed an association between fatigue of arthritis and pain and challenging affective states [42]. According to Zautra et al. (2007), a past depression episode might suggest that pain at baseline was higher than in individuals without a depression history [43]. In this study, social stress boosted the effect of this relation. Research on psychosocial factors that influence the relationship between arthritis symptoms and emotional problems was noted by Covic et al. (2003), which found that powerlessness not only influences depression, but also increases perceptions of pain [44] and facilitates the connection between pain and depression. According to Chaney et al. (2004), patients’ inner characterizations, such as helplessness, increase their experiences of depression and pain [45]. Similarly, Frantom et al. (2006) concluded that psycho-social factors, such as stress and self-efficacy, have a definite association with pain [46].

Finally, inflammation associated with arthritis may be the cause of poor cognitive functions. As discussed in the introduction, inflammation is negatively related to cognitive functions in middle-aged individuals [6]. Animal studies have also found that inflammation associated with osteoarthritis may promote Alzheimer’s disease pathology [9]. However, a future study should test if this is the case.

There are many strengths of this study, which include a relatively large sample size and well controlled sociodemographic characteristics. However, there are some limitations, as well. First, pathologies, pain, treatment (e.g., anti-inflammatory medication), mental health, quality of life, and other comorbidities, such as stroke, may contribute to poor cognitive performance in people with arthritis. Future studies should assess these conditions. However, we did not control these in the current study. Second, the type of arthritis was not differentiated in our study. Future studies should include questions that assess the type of arthritis. Finally, as with all other cross-sectional studies, causal effects cannot be identified in the current research. A longitudinal strategy should be used in future investigations to determine if arthritis leads to poor cognitive performance or if poor cognitive performance is a risk factor of arthritis.

An individual with arthritis experiences various somatic symptoms, such as joint deformation, tiredness, insomnia, and weight loss, as well as a number of psychological problems, such as depression, anxiety, stress, feelings of powerlessness, and social challenges, which can impact their ability to perform everyday roles [31]. Pharmaceutical treatments, yoga, and intervention programs may be helpful to the patient. For instance, rheumatoid arthritis (RA) is commonly treated with non-steroidal anti-inflammatory drugs (NSAIDs), disease-modifying anti-rheumatic drugs (DMARDs), biological agents, and immunosuppressants [47], but their usage is limited by serious side effects. However, reducing inflammation could potentially improve cognitive performance, as inflammation is one of the factors that contribute to poor cognitive performance. Natural extracts, containing polyphenolic chemicals, have been proposed as an attractive adjuvant medicine for the overall therapy of rheumatoid arthritis [48]. Chinese herbs have also demonstrated better safety and efficacy profiles compared to chemosynthetic medications [49]. Yoga may be seen as an ancillary treatment for arthritis, as it can lead to significant improvements in handgrip strength [50], limit symptoms of depression [51], oxidative stress [52], anxiety, and reduce stress, resulting in more relaxation and better concentration [53,54], which may then increase cognitive performance, as these may be the potential factors that contribute to the relationship between arthritis and cognition. Systematic reviews have demonstrated the helpfulness of yoga for arthritis patients. Intervention programs can improve outcomes of those with disabilities, boost functionality, leading to better cognitive performance and mental health outcomes [12]. Hence, pharmaceutical treatments, yoga, and intervention programs may be helpful to the patient in managing RA symptoms.

Finally, there are several other ways of improving cognitive performance in people with arthritis. For instance, exercise can increase blood flow to the brain, reduce inflammation, and improve mood, all of which can lead to better cognitive performance [55]. Cognitive training involves exercises designed to improve cognitive function, such as memory, attention, and executive function [55]. These exercises can be performed through computer-based programs or in-person training sessions with a healthcare professional. Maintaining social connections and engaging in meaningful activities with others can help improve cognitive functions. Social engagement can also help reduce stress and improve mood, which can have a positive impact on cognitive performance. However, future studies need to test if these interventions are feasible and helpful in arthritis patients.

## 5. Conclusions

Taken together, this study provided novel findings that many cognitive domains including episodic memory, semantic verbal fluency, fluid intelligence, and numerical abilities are negatively affected by arthritis in a cohort-based study based on participants from the United Kingdom. Strengths of this study included a relatively large sample size and well controlled sociodemographic characteristics, whereas weaknesses of this study included uncontrolled comorbidities, undifferentiation of types if arthritis, and cross-sectional design. Future studies should control for other factors that may contribute to poor cognitive performance, use more objective measures, and adopt a longitudinal design to further clarify the relationships between arthritis and cognition.

## Figures and Tables

**Table 1:** Word lists for the immediate and delayed word recall tasks.

Word List 1	Word List 2	Word List 3	Word List 4
HOTEL	SKY	WOMAN	WATER
RIVER	OCEAN	ROCK	CHURCH
TREE	FLAG	BLOOD	DOCTOR
SKIN	DOLLAR	CORNER	PALACE
GOLD	WIFE	SHOES	FIRE
MARKET	MACHINE	LETTER	GARDEN
PAPER	HOME	GIRL	SEA
CHILD	EARTH	HOUSE	VILLAGE
KING	COLLEGE	VALLEY	BABY
BOOK	BUTTER	ENGINE	TABLE

**Table 2:** Descriptive statistics of demographic characteristics and cognitive performance for people with and without arthritis. S.D. = standard deviation.

	People with Arthritis		People without Arthritis	
Variables	Mean	S.D.	Mean	S.D.
Age	61.18	14.51	61.44	9.95
Monthly income	1275.32	1009.64	1449.49	1488.27
	N	%	N	%
Sex				
Male	771	34.15	1294	34.12
Female	1487	65.85	2498	65.88
Highest educational qualification				
Below college	1769	78.34	2799	73.81
College	489	21.66	993	26.19
Legal marital status				
Single	985	43.62	1345	35.47
Married	1273	56.38	2447	64.53
Residence				
Urban	732	75.64	2688	70.89
Rural	182	24.36	1104	29.11
	Mean	S.D.	Mean	S.D.
Immediate task recall (standardized)	−0.28	0.96	−0.13	0.97
Delayed task recall (standardized)	−0.29	0.96	−0.16	0.97
Semantic verbal fluency (standardized)	−0.16	1.00	−0.07	0.98
Fluid reasoning (standardized)	−0.26	0.95	−0.14	0.97
Numerical ability (standardized)	−0.14	0.88	−0.02	0.86

**Table 3:** The ANOVA results for the model trained on healthy controls for A. intermediate word recal, B. delayed word recall, C. animal naming, D. number series, and E. numeracy, respectively. SumSq = sum squared, DF = degrees of freedom, MeanSq = mean squared, F = F statistics, *p*-Value = *p* value.

**A. Immediate Word Recall**					
	SumSq	DF	MeanSq	F	*p*-Value
Age	292.25	1	292.25	379.41	*p* < 0.001
Sex	60.79	1	60.79	78.92	*p* < 0.001
Monthly Income	11.46	1	11.46	14.88	*p* < 0.001
	107.53	1	107.53	139.6	*p* < 0.001
Highest educational qualification					

Legal marital status	4.5	1	4.5	5.84	*p* < 0.05
Residence	7.1	1	7.1	9.22	*p* < 0.01
Error	2915.5	3785	0.77		
**B. Delayed word recall**					
	SumSq	DF	MeanSq	F	*p*-Value
Age	305.74	1	305.74	383.8	*p* < 0.001
Sex	60.38	1	60.38	75.79	*p* < 0.001
Monthly Income	5.15	1	5.15	6.46	*p* < 0.05
	72.72	1	72.72	91.28	*p* < 0.001
Highest educational qualification					

Legal marital status	6.1	1	6.1	7.66	*p* < 0.01
Residence	6.11	1	6.11	7.67	*p* < 0.01
Error	3015.2	3785	0.8		
**C. Animal naming**					
	SumSq	DF	MeanSq	F	*p*-Value
Age	198.86	1	198.86	233.84	*p* < 0.001
Sex	3.82	1	3.82	4.49	*p* < 0.05
Monthly Income	11.28	1	11.28	13.26	*p* < 0.001
	65.63	1	65.63	77.17	*p* < 0.001
Highest educational qualification					

Legal marital status	13.21	1	13.21	15.54	*p* < 0.001
Residence	16.18	1	16.18	19.02	*p* < 0.001
Error	3218.8	3785	0.85		
**D. Number series**					
	SumSq	DF	MeanSq	F	*p*-Value
Age	84.24	1	84.24	103.01	*p* < 0.001
Sex	20.42	1	20.42	24.97	*p* < 0.001
Monthly Income	30.73	1	30.73	37.57	*p* < 0.001
	139.31	1	139.31	170.34	*p* < 0.001
Highest educational qualification					

Legal marital status	39.5	1	39.5	48.3	*p* < 0.001
Residence	17.67	1	17.67	21.6	*p* < 0.001
Error	3095.4	3785	0.82		
**E. Numeracy**					
	SumSq	DF	MeanSq	F	*p*-Value
Age	15.22	1	15.22	23.74	*p* < 0.001
Sex	58.98	1	58.98	91.99	*p* < 0.001
Monthly Income	28.13	1	28.13	43.87	*p* < 0.001
	107.04	1	107.04	166.97	*p* < 0.001
Highest educational qualification					

Legal marital status	25.13	1	25.13	39.21	*p* < 0.001
Residence	14.48	1	14.48	22.58	*p* < 0.001
Error	2426.4	3785	0.64		

**Table 4:** The estimates (*b*) of linear models trained based on demographic predictors. All numbers are rounded up to two digits.

	Intermediate Word Recall	Delayed Word Recall	Animal Naming	Number Series	Numeracy
Age	−0.03 **	−0.03 ***	−0.02 ***	−0.01 ***	−0.01 ***
Sex	0.28 ***	0.28 ***	0.07 *	0.06 ***	−0.27 ***
Monthly income	0.00 ***	0.00 *	0.00 ***	0.00 ***	0.00 ***
Highest educational qualification	0.41 ***	0.33 ***	0.32 ***	−0.08 ***	0.41 ***
Legal marital status	0.07 *	0.09 **	0.13 ***	−0.22 ***	0.17 ***
Residence	0.10 **	0.09 **	0.14 ***	−0.03	0.14 ***
R^2^	0.18	0.16	0.11	0.14	0.14

* *p* < 0.05 ** *p* < 0.01 *** *p* < 0.001.

## Data Availability

This data can be found here: https://www.understandingsociety.ac.uk.
